# Efficacy and toxicity of different concurrent chemoradiotherapy regimens in the treatment of advanced cervical cancer: A network meta-analysis

**DOI:** 10.1097/MD.0000000000005853

**Published:** 2017-01-13

**Authors:** Zhan-Zhao Fu, Kun Li, Yong Peng, Yue Zheng, Li-Yan Cao, Yun-Jie Zhang, Yong-Mei Sun

**Affiliations:** a Department of Radiotherapy, the First Hospital of Qinhuangdao; b Yanshan University; c Department of Biomedical Engineering, Yanshan University; d The First Hospital of Qinhuangdao; e Department of Gynaecology, the First Hospital of Qinhuangdao, Qinhuangdao, P.R. China. .

**Keywords:** cervical cancer, concurrent chemoradiotherapy, efficacy, network meta-analysis, toxicity

## Abstract

**Objective:**

The aim of this study was to compare the efficacy and toxicity of different concurrent chemoradiotherapy (CCRT) regimens in the treatment of advanced cervical cancer (CC) by adopting a network meta-analysis.

**Methods:**

We searched PubMed and Cochrane Library from the inception of these databases to September 2016, and all cohort studies (CSs) related to different CCRT regimens in the treatment of CC were included. A network analysis was adopted to compare the combination of direct and indirect evidence, to analyze the odds ratio (OR), and to draw a surface under the cumulative ranking curve of the efficacy and toxicity of different CCRT regimens for CC. Cluster analyses were used to group each category based on similar treatment regimens.

**Results:**

Nineteen CSs were enrolled in this network meta-analysis, including 12 CCRT regimens (radiotherapy [RT], CCRT [cisplatin], CCRT [vinorelbine], CCRT [paclitaxel], CCRT [hydroxyurea], CCRT [cisplatin + FU], CCRT [cisplatin + gemcitabine], CCRT [cisplatin + docetaxel], CCRT [cisplatin + paclitaxel], CCRT [cisplatin + amifostine], CCRT [cisplatin + FU + hydroxyurea], and CCRT [cisplatin + vincristine + bleomycin]). The results of the network meta-analysis showed that regarding efficacy, the overall response rate of CCRT (cisplatin + docetaxel) was higher than RT, and the 5-year overall survival (OS) rate of CCRT (cisplatin + FU + hydroxyurea) was relatively higher than CCRT (hydroxyurea). As for toxicity, CCRT (cisplatin) had a lower incidence of leukopenia than CCRT (hydroxyurea), CCRT (cisplatin + FU) and CCRT (cisplatin + paclitaxel), and the incidences of diarrhea and vomiting in CCRT (cisplatin) were lower than those in CCRT (cisplatin + gemcitabine). Additionally, the cluster analysis showed that CCRT (cisplatin) had relatively lower incidences of both hematotoxicity and gastrointestinal toxicity, and CCRT (paclitaxel) had lower gastrointestinal toxicity than other regimens.

**Conclusion:**

Our study demonstrated that CCRT (cisplatin + docetaxel) might be the best choice of CCRT regimens in the treatment of CC, and the 5-year OS rate of CCRT (cisplatin + FU + hydroxyurea) might be the highest among these different regimens. CCRT (cisplatin) might have the lowest toxicity among all the CCRT regimens.

## Abstract 2

Supplemental Digital Content is available in the text

## 1 Introduction

Cervical cancer (CC) is the second most common female cancer worldwide.^[1]^ In many developing countries, CC remains a major public health problem with high overall incidence and a higher frequency of advanced stage at diagnosis.^[2]^ Lack of awareness, the low level of effective screening programs, overshadowing by other health priorities (such as acquired immune deficiency syndrome, tuberculosis, and malaria), and insufficient attention to women's health are the possible factors for the observed higher incidence rate of CC.^[3]^ Even as a result of screening, most cervical cancers could be identified early and cured with surgery, but the lack of routine population-based screening in some parts of the word results in the majority of unscreened patients presenting with locally advanced CC.^[4]^ Pelvic radiotherapy (RT) and intracavitary brachytherapy used to be the main treatment modality for patients with advanced CC and has played an important role in the treatment of CC.^[5]^ The results of RT depend on disease stage, tumor volume, the presence of involved lymph nodes, delivered radiation dose, treatment duration, hemoglobin level, and the optimal use of intracavitary brachytherapy.^[2]^ Furthermore, the major limitation to reaching a curative radiation dose in tumors is the high sensitivity to radiation and subsequent damage to the surrounding normal tissues.^[6]^ Thus, the prognosis of patients with advanced CC is still poor, particularly for those with bulky local tumors and extensive parametrical extension.^[7]^ Nodal involvement, particularly of paraaortic nodes, was reported to be the most important adverse prognostic factor of reduced survival rate for advanced CC.^[2]^ Nevertheless, a series of studies has shown that the outcome of advanced CC patients can be improved by the use of concurrent chemoradiotherapy (CCRT) regimens.

CCRT is the standard organ-preservation treatment for resectable disease; it ensures optimal locoregional control and has become a cornerstone of treatment.^[8]^ The current traditional treatment of choice for advanced CC has been CCRT.^[9]^ CCRT can improve pelvic control and survival in advanced CC patients because it can provide active systemic cytotoxic agents against CC with the potential to enhance radiosensitivity and local tumor control and to eradicate micrometastasis.^[10]^ A chemotherapeutic drug, Cisplatin, was reported to be one of the most effective agents and is widely used in the treatment of CC.^[11]^ It has been demonstrated in previous randomized trials that in the last decade the results of RT are significantly improved with the addition of cisplatin-based CCRT and have become the standard of care; consequently, cisplatin-based CCRT was rapidly adopted in clinical practice for the treatment of advanced CC.^[12]^ Although cisplatin-based CCRT was considered standard treatment for advanced CC, it could be difficult to use in aged patients or patients with comorbidities such as diabetes mellitus and hypertension.^[13]^ However, better results have showed that gemcitabine with cisplatin-based CCRT followed by 2 cycles of adjuvant gemcitabine and cisplatin is a cost-effective treatment for locally advanced CC.^[14]^ It is almost certain that there are many other effective CCRT regimens for the treatment of advanced CC, but which is the best one?

Network meta-analysis can provide estimates of multiple treatment regimens, even when direct comparisons are unavailable.^[15]^ Therefore, in this article, we performed a network meta-analysis to compare the efficacy and toxicity of different CCRT regimens to investigate which is the best choice in the treatment of advanced CC.

## 2 Methods

### 2.1 Electronic searches

Computer-based retrieval of PubMed and the Cochrane Library databases (from inception to September 2016), combined with manual retrieval of related references, were performed. Combining the keywords and free words, the search terms were as follows: Concurrent chemoradiotherapy (CCRT), cohort study (CS), and cervical cancer.

### 2.2 Data collection and analysis

Studies were included in this network meta-analysis if they met the following criteria: the study design must be CSs; the study subjects should be patients with advanced CC aged 18 to 87 years; the end outcomes of studies should include efficacy (overall response rate [ORR], 5-year overall survival [OS] rate, 5-year disease-free survival [DFS] rate) or toxicity (anemia, leukopenia, neutropenia, thrombocytopenia, diarrhea, nausea, vomiting). Studies were excluded if the following criteria were met: patients were previously treated with pelvic radiotherapy or systemic chemotherapy; patients were pregnant or lactating; patients had severe or uncontrolled infection or other uncontrolled systemic diseases; the studies contained incomplete literature data or were non-CSs, duplications, conference reports, meta-analyses, abstracts or non-English publications. The present study is a network meta-analysis, and the eligible patients included in our study were obtained from our included studies; therefore, the ethics statement in the present study was waived based on this case.

### 2.3 Data extraction and quality assessment

Two researchers extracted the data of included studies independently. Additionally, a third researcher was consulted if agreement could not be reached between these 2 researchers. Two or more researchers reviewed the CSs according to Newcastle-Ottawa Scale (NOS).^[16]^ The NOS assigns up to a maximum of 9 points for the least risk of bias in 3 domains: selection of CS (whether the exposed CSs are representative [NOS1], whether the non-exposed CSs are drawn from the same community as the exposed CSs [NOS2], whether the CSs have a secure record or structured interview [NOS3], whether the outcome of interest was present at the start of the study [NOS4]); comparability of CS (whether the CSs were selected or controlled based on the most important factor [NOS5], and whether the CSs were controlled for any additional factor [NOS6]; ascertainment of exposure and outcomes for CSs (whether the assessment of outcome was independent and blind [NOS7], whether the follow-up period for outcomes to occur was long enough [NOS8], whether all subjects completely followed-up or subjects lost to follow-up were unlikely to introduce bias [NOS9]). CSs with points of ≥5 were included in this network meta-analysis.

### 2.4 Statistical methods

Traditional pairwise meta-analyses were adopted to directly compare the different CCRT regimens. The odd ratios (ORs) or weighted mean differences (WMDs) with their 95% confidence interval (95% CI) were calculated under a fixed effects model or random effects model. The significance of the combined effect was detected by the *Z* test.^[17]^ The heterogeneity of these regimens was evaluated using Cochran's Q-statistic and the *I*^*2*^ test among the enrolled studies.^[18,19]^ When *P*_*h*_ > 0.05 or *I*^*2*^ < 50%, heterogeneity was indicated, and the fixed effects model was used; if the converse was true, the random effects model was adopted.^[20]^ A network evidence plot was drawn with the nodes indicating interventions, the node size representing sample sizes, and the thickness of lines referring to the accuracy of the effect size of the comparison between 2 studies (the inverse of variance). A surface under the cumulative ranking (SUCRA) curve was used to compare the SUCRA value of different CCRT regimens to ascertain the efficacy and toxicity of the different chemotherapy regimens; the larger the SUCRA value, the better the treatment or the lower the toxicity.^[21]^ Cluster analyses were adopted to compare the efficacy and toxicity of different CCRT regimens in the treatment of advanced CC, according to the similarity of 2 variables to cluster the different interventions, and different intervention measures were determined to judge the merits of the effect.^[21]^ The presence of a small-study effect was assessed using a comparison-adjusted funnel plot, which takes into account different summary effects for each set of studies (measure of precision vs. estimated treatment effect).^[22]^ All computations were performed using Stata 13.1 (Corp, College Station, TX) software.

## 3 Results

### 3.1 Characteristics of the included studies

**Figure 1:**
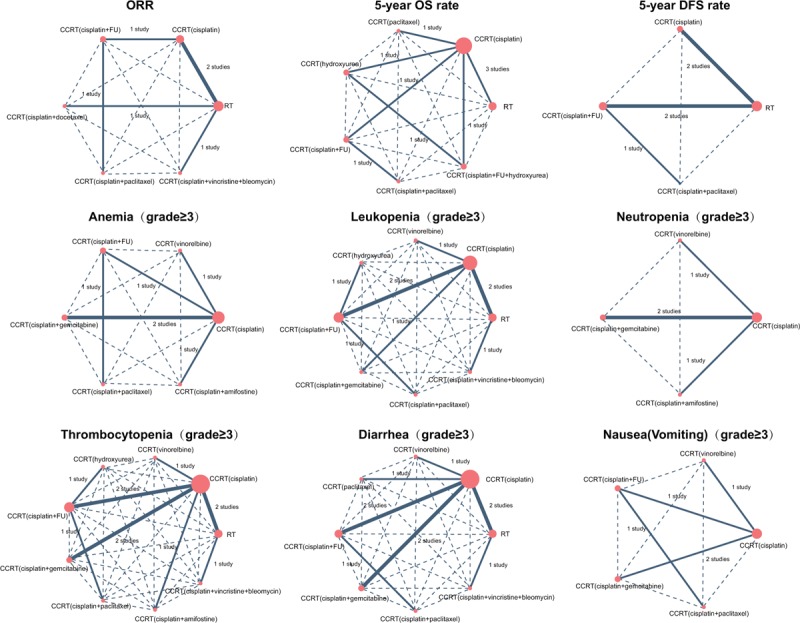
Evidence plots of the ORR, OS, and DFS of the 12 CCRT regimens included in this network meta-analysis. CCRT = concurrent chemoradiotherapy, DFS = disease-free survival, ORR = overall response rate, OS = overall survival.

A total of 295 publications were initially retrieved in this study, and 5 repeated assays, 109 letters or summaries, 21 non-human studies, and 17 non-English articles were eliminated. Moreover, 260 non-CSs, 321 articles unrelated to advanced CC, 58 articles unrelated to CCRT, and 1 article without data or incomplete data were also rejected from the remaining 143 assays. Nineteen CSs met the inclusion criteria and were eventually selected into our meta-analysis from 1989 to 2015^[2,7,13,23–38]^ (Supplementary Figure 1). A total of 3170 patients with advanced CC were enrolled in these studies, including 12 CCRT regimens (RT, CCRT [cisplatin], CCRT [vinorelbine], CCRT [paclitaxel], CCRT [hydroxyurea], CCRT [cisplatin + FU], CCRT [cisplatin + gemcitabine], CCRT [cisplatin + docetaxel], CCRT [cisplatin + paclitaxel], CCRT [cisplatin + amifostine], CCRT [cisplatin + FU + hydroxyurea], and CCRT [cisplatin + vincristine + bleomycin]) (Fig. 1). Ten included studies were performed in whites, and the other RCT was performed in Asians. Furthermore, 18 included studies were conducted as a 2-arm trial, and the other was a 3-arm trial. The characteristics of the included studies were summarized in Table 1,^[2,7,13,23–38]^ and a NOS quality assessment is shown in Supplementary Figure 2.

**Table 1:**
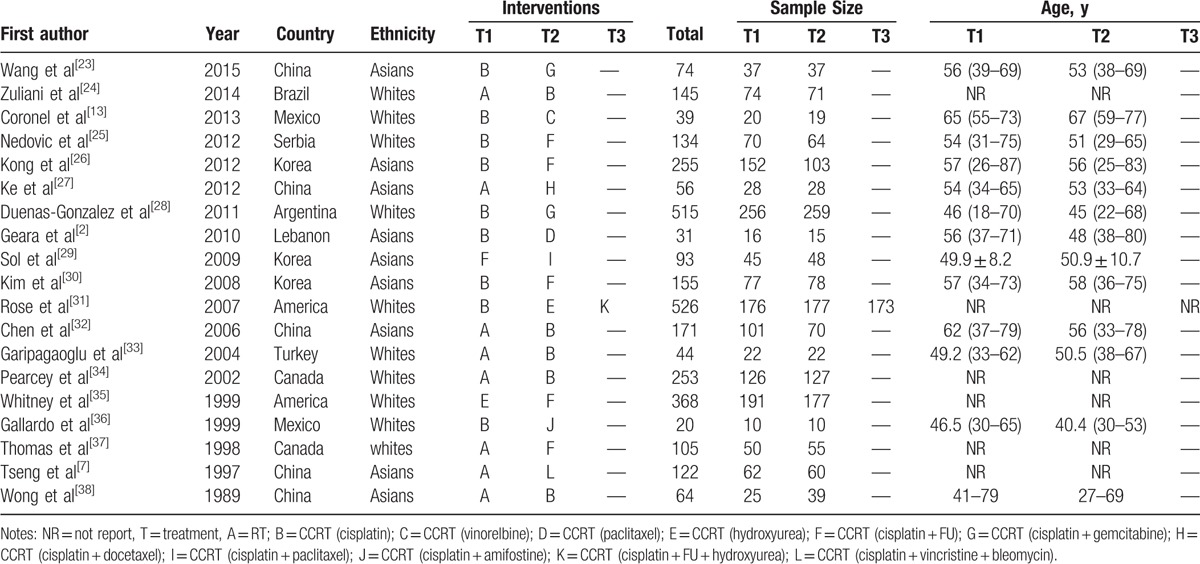
The baseline characteristics for included studies.

### 3.2 Pairwise meta-analysis of the efficacy and toxicity of different CCRT regimens

The results of pairwise meta-analysis showed that in terms of efficacy, the 5-year OS rates of CCRT (cisplatin) and CCRT (cisplatin + FU + hydroxyurea) were relatively higher than CCRT (hydroxyurea); however, there were no significant differences in the ORR and 5-year DFS rate among these 12 CCRT regimens (Table 2). As for hematotoxicity, compared with CCRT (cisplatin), the incidences of anemia, neutropenia, and thrombocytopenia with CCRT (cisplatin + gemcitabine) were relatively higher; compared with CCRT (cisplatin + FU), the incidences of leukopenia with CCRT (hydroxyurea) and CCRT (cisplatin + paclitaxel) were relatively higher. As far as gastrointestinal toxicity, compared with CCRT (cisplatin), the incidence of diarrhea with CCRT (cisplatin + gemcitabine) was higher, and the incidence of nausea with CCRT (cisplatin + FU) was relatively higher; however, there was no significant difference of the incidence of vomiting among these 12 CCRT regimens (Appendix Table 1).

**Table 2:**
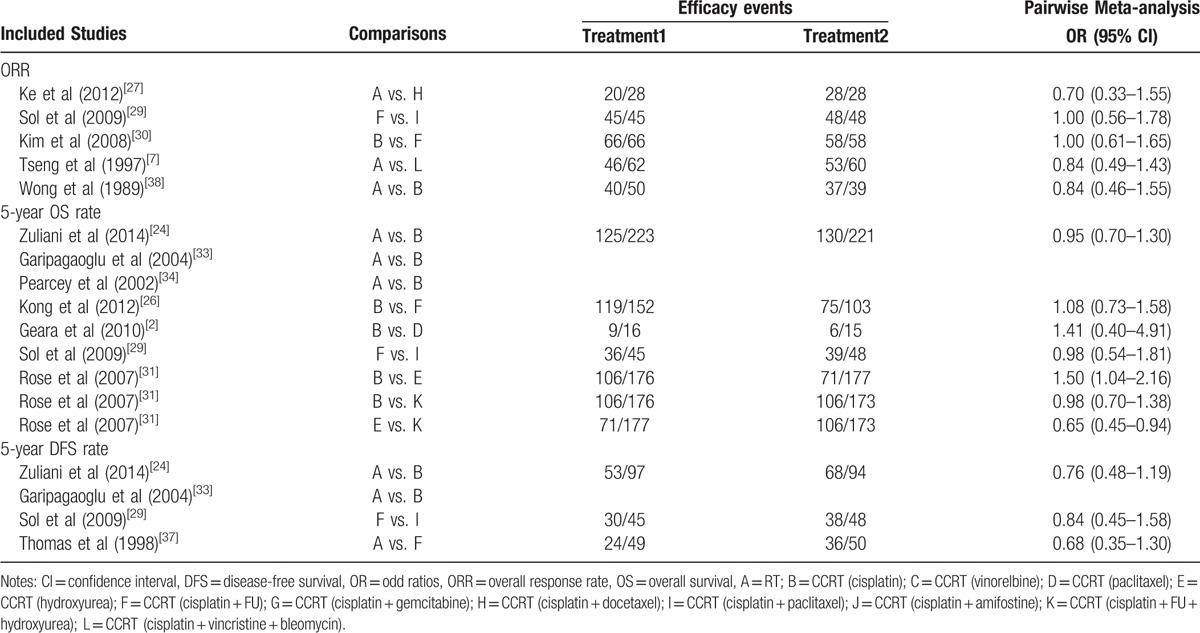
Pairwise meta-analysis for efficacy events in advanced cervical cancer patients.

### 3.3 Network meta-analysis of the efficacy and toxicity of different CCRT regimens

The network meta-analysis showed that in terms of the efficacy, the ORR of CCRT (cisplatin + docetaxel) was higher than RT (OR = 23.63, 95% CI = 1.29∼433.02); compared with CCRT (hydroxyurea), the 5-year OS rate of CCRT (cisplatin + FU + hydroxyurea) was relatively higher (OR = 2.36, 95% CI = 1.54∼3.63). Compared with RT and CCRT (cisplatin), the 5-year OS rate of CCRT (hydroxyurea) was relatively lower (OR = 0.49, 95% CI = 0.28∼0.87; OR = 0.44, 95% CI = 0.29∼0.68, respectively); however, there was no significant difference of 5-year DFS rate among these 12 regimens.

In the case of hematotoxicity, compared with CCRT (cisplatin), the incidences of leukopenia with CCRT (hydroxyurea), CCRT (cisplatin + FU) and CCRT (cisplatin + paclitaxel) were relatively higher (OR = 21.30, 95% CI = 6.44∼70.45; OR = 2.42, 95% CI = 1.08∼5.44; OR = 73.59, 95% CI = 17.86∼303.31, respectively), and the incidence of thrombocytopenia with CCRT (cisplatin + gemcitabine) was higher (OR = 4.44, 95% CI = 1.63∼12.07). These results indicated that the hematotoxicity of CCRT (cisplatin) was lower than other CCRT regimens. In terms of gastrointestinal toxicity, the incidence of diarrhea and vomiting with CCRT (cisplatin + gemcitabine) was higher than CCRT (cisplatin) (OR = 3.80, 95%CI = 2.08∼6.95; OR = 2.94, 95%CI = 1.26∼6.86, respectively), and this result indicated that CCRT (cisplatin) has a lower incidence of gastrointestinal toxicity than other CCRT regimens. Compared with RT, the incidence of nausea with CCRT (cisplatin + FU) was relatively higher (OR = 2.53, 95%CI = 1.13∼5.66). However, regarding the incidence of anemia and neutropenia, there was no significant difference among these twelve CCRT regimens (Table 3, Supplementary Figure 3).

**Table 3:**
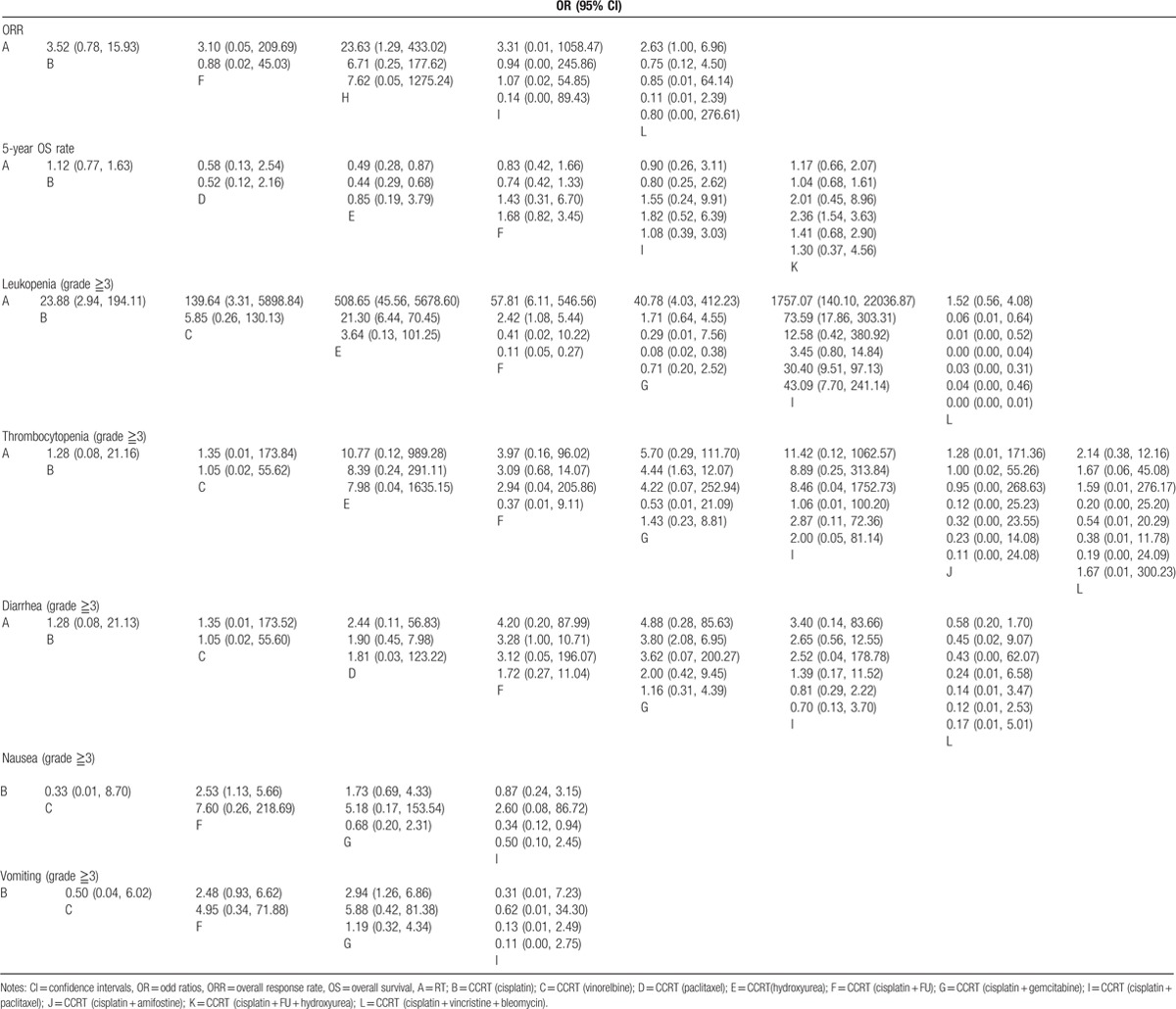
ORs and 95% CIs of 11 treatment modalities in terms of efficacy and toxicity outcomes.

### 3.4 Cumulative probability of the efficacy and toxicity of different CCRT regimens

As shown in Table 4, the SUCRA values of the efficacy and toxicity of 12 CCRT regimens demonstrated that in terms of efficacy, the ORR of CCRT (cisplatin + Docetaxel) ranked the highest (85.7%); the 5-year OS rate of CCRT (cisplatin + FU + hydroxyurea) ranked the highest (76.4%); and the 5-year DFS rate of CCRT (cisplatin + paclitaxel) ranked the highest (81.5%). As for toxicity, the incidence of anemia and nausea with CCRT (cisplatin + FU) ranked the lowest (anemia: 34.0%; nausea: 10.2%), whereas the incidence of neutropenia, diarrhea, and vomiting of CCRT (cisplatin + gemcitabine) ranked the lowest (neutropenia: 20.4%; diarrhea: 20.5%; vomiting: 14.6%). The incidence of leukopenia and thrombocytopenia of CCRT (cisplatin + paclitaxel) ranked the lowest (leukopenia: 1.8%; thrombocytopenia: 25.6%).

**Table 4:**
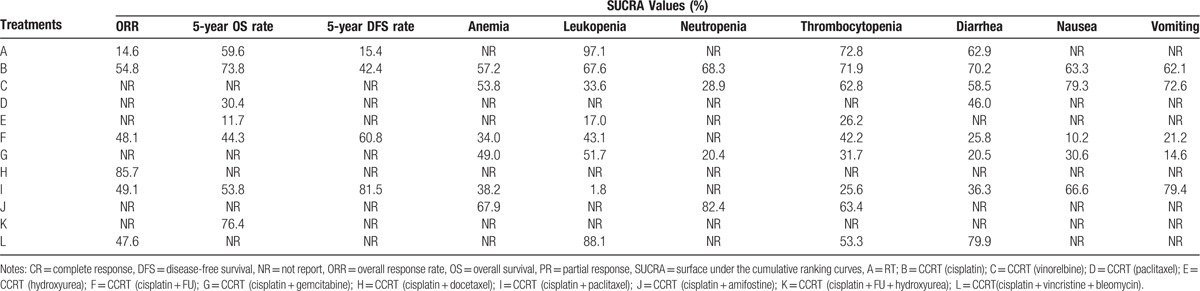
SUCRA values of 12 treatment modalities under 10 endpoint outcomes.

### 3.5 Cluster analysis and publication bias regarding efficacy and toxicity in the included studies

**Figure 2:**
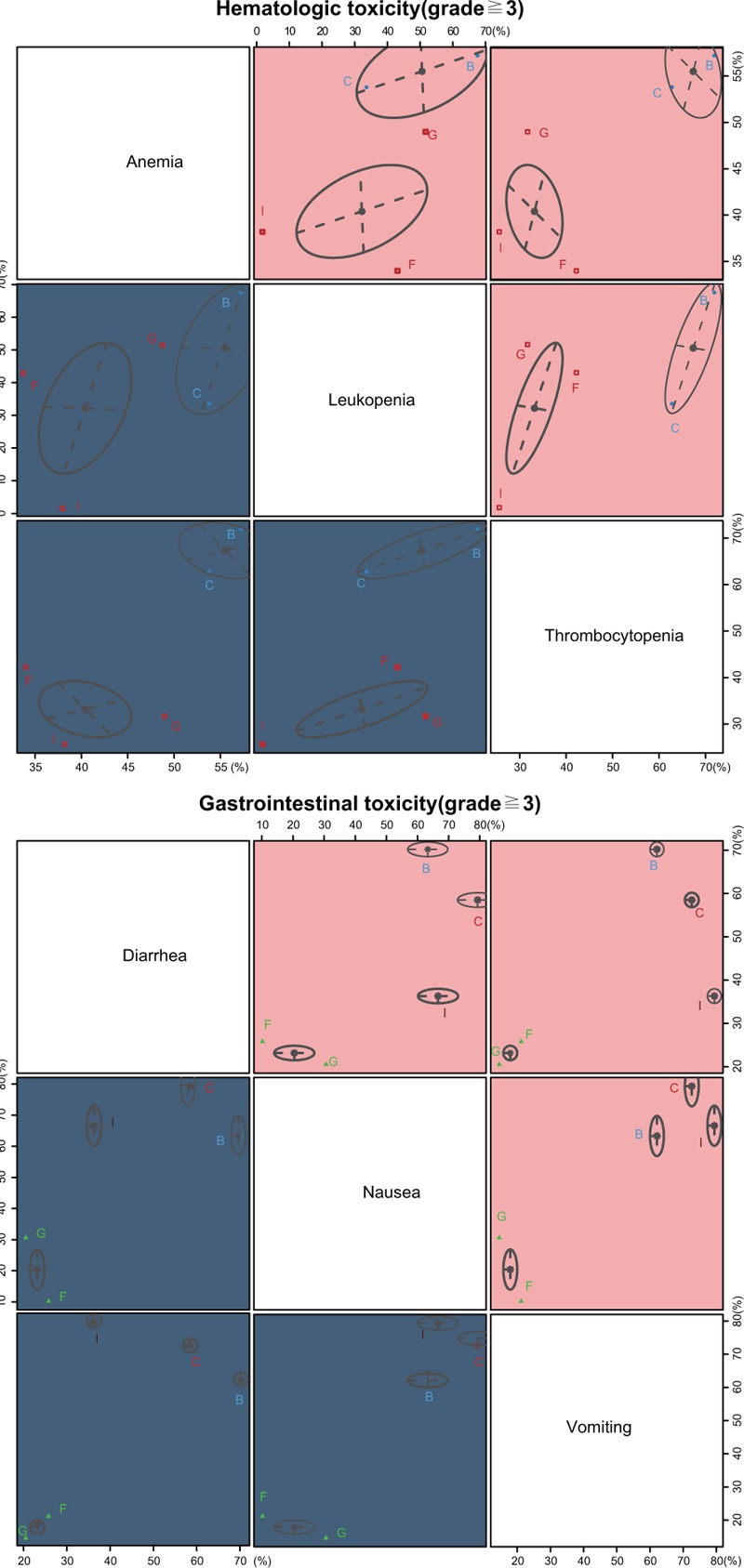
Clustered ranking plots based on SUCRA values of the efficacy (ORR, 5-year OS rate, 5-year DFS rate) and toxicity (anemia, leukopenia, neutropenia, thrombocytopenia, diarrhea, nausea, and vomiting) of the 12 CCRT regimens in the treatment of advanced CC. CCRT = concurrent chemoradiotherapy, DFS = disease-free survival, ORR = overall response rate, OS = overall survival, SUCRA = surface under the cumulative ranking.

**Figure 3:**
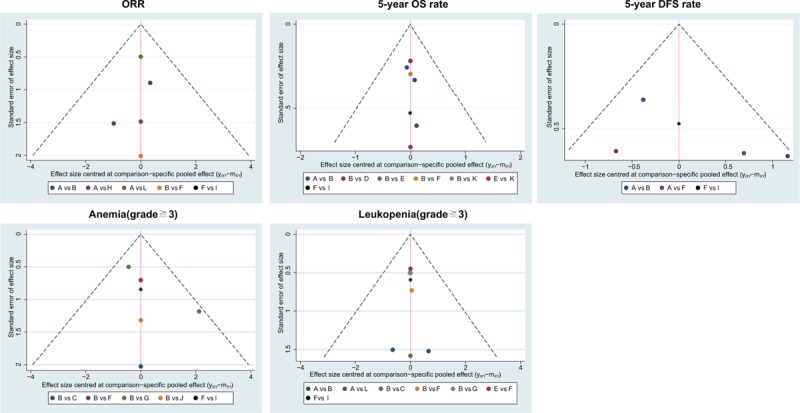
Comparison-adjusted funnel plots of the efficacy (ORR, 5-year OS rate and 5-year DFS rate) and toxicity (anemia and leukopenia) of the 12 CCRT regimens. CCRT = concurrent chemoradiotherapy, DFS = disease-free survival, ORR = overall response rate, OS = overall survival.

**Figure 4:**
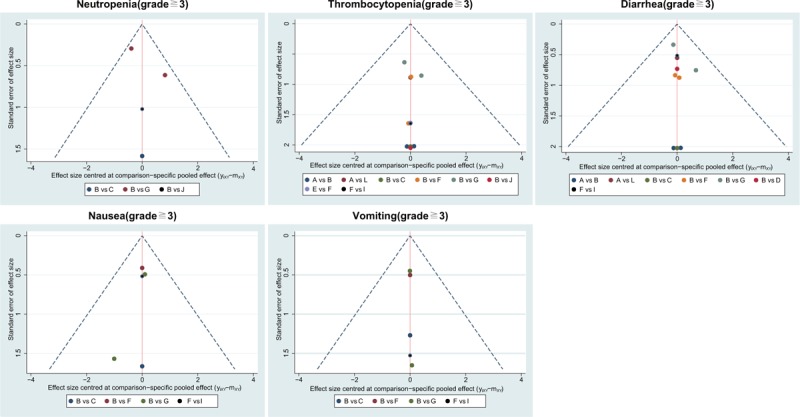
Comparison-adjusted funnel plots of the toxicity (neutropenia, thrombocytopenia, diarrhea, nausea, and vomiting) of the 12 CCRT regimens in the treatment of advanced CC. CC = cervical cancer, CCRT = concurrent chemoradiotherapy,

The cluster analysis based on SUCRA values indicated that the CCRT (cisplatin) regimen had lower hematotoxicity than others, wheraes CCRT (cisplatin) and CCRT (vinorelbine) had lower gastrointestinal toxicity than other CCRT regimens, and CCRT (cisplatin + FU) and CCRT (cisplatin + gemcitabine) had relatively higher gastrointestinal toxicity (Fig. 2). The comparison-adjusted funnel plot of the efficacy (ORR, 5-year OS rate, 5-year DFS rate) and toxicity (anemia, leukopenia, neutropenia, thrombocytopenia, diarrhea, nausea, and vomiting) of these 12 CCRT regimens showed that there was no publication bias among the included studies (Figs. 3 and 4).

## 4 Discussion

Our network meta-analysis included 19 cohort studies with a total of 3170 advanced CC patients, most of which were related to the comparison of RT versus CCRT (cisplatin), and the majority of these advanced CC patients were treated by CCRT (cisplatin). The main results of our study showed that the short-term efficacy of CCRT (cisplatin + docetaxel) was better than other CCRT regimens. CCRT with low-dose cisplatin was tolerable and showed a favorable initial response as the primary therapy for advanced CC.^[39]^ Cisplatin, as an assistant drug for chemotherapy, was often combined with other drugs to treat cancers but was also accompanied by some side effects.^[40]^ In our study, the toxicity (both hematotoxicity and gastrointestinal toxicity) of CCRT (cisplatin) was relatively lower than the other included CCRT regimens. Additionally, FU is commonly used to treat skin cancer or damage caused by the HPV virus so that it benefitted CC caused by the HPV virus.^[41]^ Previous studies had also confirmed that if FU was used alone, the curative rate was approximately 75%; therefore, it was better to combine it with other drugs.^[42]^ This supported our finding that the 5-year OS rate of CCRT (cisplatin + FU + hydroxyurea) was relatively higher than the other CCRT regimens in our study.

The pairwise meta-analysis of our study showed that the incidences of anemia, neutropenia, and thrombocytopenia with CCRT (cisplatin + gemcitabine) were higher than CCRT (cisplatin). However, the incidence of diarrhea with CCRT (cisplatin + gemcitabine) and the incidence of nausea with CCRT (cisplatin + FU) was higher than CCRT (cisplatin). Gemcitabine is a poly-ADP-ribose polymerase (PARP) inhibitor that could inhibit the proliferation of tumor cells by blocking PARP signaling.^[43]^ Furthermore, gemcitabine can inhibit the growth of cancer cells by targeting particular factors.^[44]^ A previous study indicated that FU had some toxicity and side effects that could cause body pain and endothelial tissue ulceration; at the same time, its characteristic long-term onset time makes it easier for patients to develop drug resistance.^[42,45]^

Additionally, the SUCRA values of the efficacy and toxicity of twelve CCRT regimens demonstrated that the ORR with CCRT (cisplatin + docetaxel) was higher than other CCRT regimens. The incidence of anemia and nausea with CCRT (cisplatin + FU) was higher than other CCRT regimens, whereas the incidence of leukopenia and thrombocytopenia with CCRT (cisplatin + paclitaxel) was higher than other CCRT regimens. A randomized phase III trial demonstrated that cisplatin + docetaxel also had superior response rates and survival in the treatment of previously untreated patients with stage IV non-small-cell lung cancer.^[46]^ McGuire et al^[47]^ suggested that incorporating paclitaxel into first-line therapy also can improve the duration of progression-free survival and overall survival in women with incompletely resected stage III and stage IV ovarian cancer.

In conclusion, our study demonstrated that CCRT (cisplatin + docetaxel) might be the best choice of CCRT regimens in the treatment of CC; moreover, the 5-year OS rate of CCRT (cisplatin + FU + hydroxyurea) might be the highest among these different regimens, and CCRT (cisplatin) might have the lowest toxicity among all the CCRT regimens. These findings may help clinicians in their choice of proper chemotherapy regimens for patients with CC. However, our network meta-analysis included only 19 cohort studies with 13 CCRT regimens. This showed that there was no remarkable long-term benefit for patients’ survival or local disease control of the included CCRT regimens in this study. And the 19 enrolled studies both including cohort studies and randomized controlled trails, which may affect the reliability and application value of our results because of the level of evidence for these enrolled studies was different. Therefore, the incidence of late intestinal toxicity still requires further investigation, and more and more randomized controlled trails about the efficacy and toxicity of different CCRT regimens in treating advanced CC are supposed to be conducted.

## Acknowledgments

The authors want to thank for all the people for their help in the paper editing.

## Supplementary Material

Supplemental Digital Content
