# Supplementary material for: Efficacy and toxicity of different concurrent chemoradiotherapy regimens in the treatment of advanced cervical cancer: A network meta-analysis
Source: Medicine (Baltimore). 2017 Jan 13;96(2):e5853. doi: 10.1097/MD.0000000000005853 (PMC5266181; doi:10.1097/MD.0000000000005853)
Supplement: Supplemental Digital Content [file medi-96-e5853-s001.doc]

**Supplementary Figure 1** Flowchart showing the literature search and study selection. Nineteen clinical cohort studies met the inclusion criteria were included in this network meta-analysis.

**Supplementary Figure 2** Newcastle-Ottawa Scale of the quality of included studies.

**Supplementary Figure 3** Confidence intervals for the 5-year DFS rate, anemia and neutropenia of the 12 CCRT regimens. Note: A = RT; B = CCRT (cisplatin); C = CCRT (vinorelbine); F = CCRT (cisplatin + FU); G = CCRT (cisplatin + gemcitabine); H = CCRT (cisplatin + Docetaxel); I = CCRT (cisplatin + paclitaxel); J = CCRT (cisplatin + amifostine).


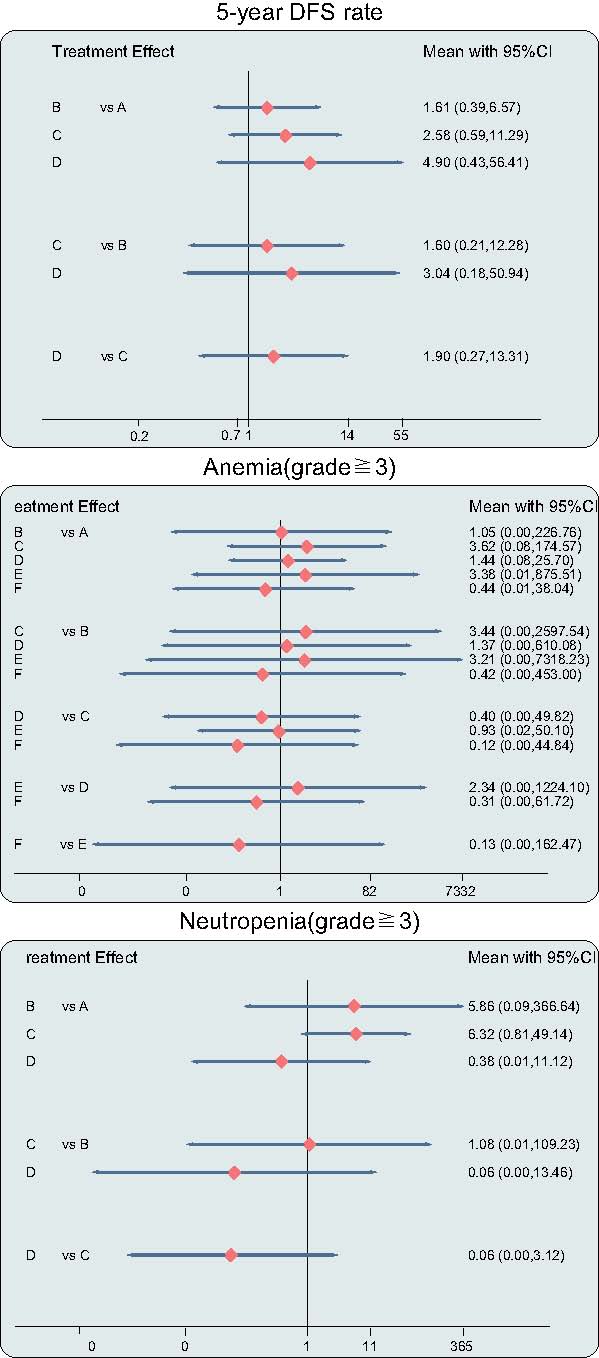


**Appendix Table 1.** Pairwise meta-analysis for toxicity events in advanced cervical cancer patients.

| **Included studies** | **Comparisons** | **Toxicity events** | |  | **Pairwise meta-analysis** |
| --- | --- | --- | --- | --- | --- |
|  |  | **Treatment1** | **Treatment2** |  | **OR (95%CI)** |
| **Hematologic toxicity(grade≧3)** | | | | | |
| **Anemia** | | | | | |
| Wang CC(2015) ^23^ | B vs. G | 8/287 | 25/296 |  | **0.33 (0.15-0.75)** |
| Duenas-Gonzalez A(2011) ^28^ | B vs. G |  |  |  |  |
| Coronel JA(2013) ^13^ | B vs. C | 1/20 | 1/19 |  | 0.95 (0.06-16.29) |
| Kong TW(2012) ^26^ | B vs. F | 3/152 | 7/103 |  | 0.29 (0.07-1.15) |
| Sol ES(2009) ^29^ | F vs. I | 3/45 | 3/48 |  | 1.07 (0.20-5.56) |
| Gallardo D(1999) ^36^ | B vs. J | 2/10 | 1/10 |  | 2.00 (0.16-25.75) |
| **Leukopenia** | | | | | |
| Wang CC(2015) ^23^ | B vs. G | 11/32 | 17/36 |  | 0.73 (0.30-1.78) |
| Coronel JA(2013) ^13^ | B vs. C | 0/20 | 2/19 |  | 0.19 (0.01-4.22) |
| Nedovic J(2012) ^25^ | B vs. F | 10/222 | 17/167 |  | **0.44 (0.20-0.99)** |
| Kong TW(2012) ^26^ | B vs. F |  |  |  |  |
| Sol ES(2009) ^29^ | F vs. I | 5/45 | 38/48 |  | **0.14 (0.05-0.39)** |
| Whitney CW(1999) ^35^ | E vs. F | 46/188 | 6/169 |  | **6.89 (2.87-16.54)** |
| Tseng CJ(1997) ^7^ | A vs. L | 8/62 | 11/60 |  | 0.70 (0.26-1.87) |
| Wong LC(1989) ^38^ | A vs. B | 0/50 | 12/39 |  | **0.06 (0.01-0.47)** |
| **Neutropenia** | | | | | |
| Wang CC(2015) ^23^ | B vs. G | 20/287 | 143/296 |  | **0.15 (0.09-0.24)** |
| Duenas-Gonzalez A(2011) ^28^ | B vs. G |  |  |  |  |
| Coronel JA(2013) ^13^ | B vs. C | 0/20 | 2/19 |  | 0.19 (0.01-4.22) |
| Gallardo D(1999) ^36^ | B vs. J | 4/10 | 2/10 |  | 2.00 (0.30-13.51) |
| **Thrombocytopenia** | | | | | |
| Wang CC(2015) ^23^ | B vs. G | 5/287 | 22/296 |  | **0.24 (0.09-0.64)** |
| Duenas-Gonzalez A(2011) ^28^ | B vs. G |  |  |  |  |
| Coronel JA(2013) ^13^ | B vs. C | 1/20 | 1/19 |  | 9.50 (0.41-217.61) |
| Nedovic J(2012) ^25^ | B vs. F | 2/222 | 5/167 |  | 0.33 (0.07-1.50) |
| Kong TW(2012) ^26^ | B vs. F |  |  |  |  |
| Sol ES(2009) ^29^ | F vs. I | 0/45 | 1/48 |  | 0.36 (0.01-8.95) |
| Whitney CW(1999) ^35^ | E vs. F | 1/188 | 0/169 |  | 2.70 (0.11-66.67) |
| Gallardo D(1999) ^36^ | B vs. J | 1/10 | 1/10 |  | 1.00 (0.05-18.30) |
| Tseng CJ(1997) ^7^ | A vs. L | 2/62 | 4/60 |  | 0.48 (0.09-2.74) |
| Wong LC(1989) ^38^ | A vs. B | 2/50 | 2/39 |  | 0.77 (0.10-5.75) |
| **Gastrointestinal toxicity(grade≧3)** | | | | | |
| **Diarrhea** | | | | | |
| Wang CC(2015) ^23^ | B vs. G | 15/287 | 52/296 |  | **0.30 (0.16-0.54)** |
| Duenas-Gonzalez A(2011) ^28^ | B vs. G |  |  |  |  |
| Coronel JA(2013) ^13^ | B vs. C | 1/20 | 1/19 |  | 0.95 (0.06-16.29) |
| Nedovic J(2012) ^25^ | B vs. F | 4/222 | 10/167 |  | 0.32 (0.10-1.04) |
| Kong TW(2012) ^26^ | B vs. F |  |  |  |  |
| Geara FB(2010) ^2^ | B vs. D | 6/16 | 8/15 |  | 0.70 (0.20-2.51) |
| Sol ES(2009) ^29^ | F vs. I | 10/45 | 9/48 |  | 1.19 (0.44-3.18) |
| Tseng CJ(1997) ^7^ | A vs. L | 10/62 | 6/60 |  | 1.61 (0.55-4.71) |
| Wong LC(1989) ^38^ | A vs. B | 2/50 | 2/39 |  | 0.77 (0.10-5.75) |
| **Nausea** | | | | | |
| Wang CC(2015) ^23^ | B vs. G | 7/287 | 13/296 |  | 0.57 (0.23-1.42) |
| Duenas-Gonzalez A(2011) ^28^ | B vs. G |  |  |  |  |
| Coronel JA(2013) ^13^ | B vs. C | 1/20 | 0/19 |  | 2.85 (0.11-74.34) |
| Kong TW(2012) ^26^ | B vs. F | 11/152 | 17/103 |  | **0.44 (0.20-0.97)** |
| Sol ES(2009) ^29^ | F vs. I | 15/45 | 7/48 |  | 2.29 (0.85-6.12) |
| **Vomiting** | | | | | |
| Wang CC(2015) ^23^ | B vs. G | 7/287 | 21/296 |  | 0.36 (0.15-0.84) |
| Coronel JA(2013) ^13^ | B vs. C | 2/20 | 1/19 |  | 1.90 (0.16-22.72) |
| Kong TW(2012) ^26^ | B vs. F | 7/152 | 11/103 |  | 0.43 (0.16-1.15) |
| Sol ES(2009) ^29^ | F vs. I | 3/45 | 0/48 |  | 7.46 (0.37-148.48) |

Notes: OR=odd ratios; CI=confidence intervals; A=RT; B=CCRT(cisplatin); C=CCRT(vinorelbine); D=CCRT(paclitaxel); E=CCRT(hydroxyurea); F=CCRT(cisplatin+FU); G=CCRT(cisplatin+gemcitabine); H=CCRT(cisplatin+docetaxel); I=CCRT(cisplatin+paclitaxel); J=CCRT(cisplatin+amifostine); K=CCRT(cisplatin+FU+hydroxyurea); L =CCRT(cisplatin+vincristine+bleomycin).
